# The role of transcriptional repressor activity of LexA in salt-stress responses of the cyanobacterium *Synechocystis* sp. PCC 6803

**DOI:** 10.1038/s41598-020-74534-7

**Published:** 2020-10-15

**Authors:** Kosuke Takashima, Syota Nagao, Ayumi Kizawa, Takehiro Suzuki, Naoshi Dohmae, Yukako Hihara

**Affiliations:** 1 Graduate School of Science and Engineering, Saitama University, 255 Shimo-Okubo, Saitama, 338-8570 Japan; 2 Biomolecular Characterization Unit, RIKEN Center for Sustainable Resource Science, 2-1 Hirosawa, Wako, Saitama 351-0198 Japan; 3 Present Address: Department of Biological Sciences, Faculty of Science and Engineering, Chuo University, 1-13-27 Kasuga, Bunkyo-ku, Tokyo, 112-8551 Japan

**Keywords:** Microbiology, Molecular biology, Plant sciences

## Abstract

Different from typical LexA repressors in heterotrophic bacteria exerting SOS response by auto-cleavage, cyanobacterial LexAs, especially that of *Synechocystis* sp. PCC 6803 (S.6803), have been suggested be involved in regulation of a number of genes related to various cellular processes, rather than the typical SOS regulon. When and how cyanobacterial LexAs are triggered to regulate its target genes have remained unknown. In this study, we found the profound repressing effect of LexA on salt-stress inducible genes in S*.*6803. The repressing activity of LexA was likely to persist during salt stress and the salt response of these genes was mainly achieved by other regulators than LexA, suggesting that the physiological role of LexA is fine-tuning of gene expression in response to environmental changes. Although the amount and oligomeric state of LexA were unchanged upon salt stress, two-dimensional electrophoresis and liquid chromatography-tandem mass spectrometry analyses detected a change in posttranslational modification in a small fraction of LexA molecules, possibly dephosphorylation of Ser^173^, after 30 min upon the upshift in salt concentration. Activity of LexA in S.6803 may be under gradual control by posttranslational modification to fine-tune gene expression, which is contrasted with the digital switching-off regulation by auto-cleavage in heterotrophic bacteria.

## Introduction

The LexA transcription factor is highly conserved among bacterial species and is well-characterized in *Escherichia coli* as the key regulator of the SOS response induced by DNA damage. Under non-stress conditions, LexA represses expression of the SOS regulon including genes involved in DNA repair, replication and cell division^1–3^. Upon SOS response, LexA interacts with RecA which is activated through binding to single-stranded DNAs formed at the sites of DNA damage. This interaction changes LexA to a cleavable conformation and auto-cleavage at the Ala^84^–Gly^85^ peptide bond is carried out by the catalytic dyad Ser^119^ and Lys^156^, leading to inactivation of LexA and the derepression of SOS regulon^4–6^.

LexA is conserved also in many cyanobacterial species, but the experimental data on model cyanobacterial species have been suggesting that regulatory roles of cyanobacterial LexAs are quite different from that of heterotrophic bacteria. Global transcriptome analyses of *Synechocystis* sp. PCC 6803 (S.6803) revealed that deletion of *lexA* positively or negatively affected expression levels of a number of genes related to various cellular processes but not genes involved in DNA repair^7–9^. Furthermore, the function of S.6803 LexA as a global regulator is suggested by the fact that LexA has been isolated as a binding factor to the upstream regions of various genes such as the *hoxEFUYH* operon encoding a bidirectional hydrogenase^10,11^, *crhR* encoding an RNA helicase^12^, *sbtA* encoding a sodium-dependent bicarbonate transporter^13^ and *fab* genes encoding fatty-acid biosynthetic enzymes^14^. Analyses of the LexA-overexpressing strain of *Anabaena* sp. PCC 7120 suggested that LexA is involved in the responses to various abiotic stresses such as oxidative stress, DNA damage, carbon limitation and heavy metal stress through regulation of diverse genes^15^.

When and how cyanobacterial LexAs are triggered to regulate its target genes have remained unknown so far. In *Anabaena* sp. PCC 7120, the auto-cleavage of LexA occurs neither at physiological pH even in the presence of the activated RecA in vitro nor under SOS-response inducing conditions in vivo, such as UV-B exposure and mitomycin C treatment^16^. In the case of S.6803, LexA lacks the Ala-Gly auto-cleavage site and the serine of the Ser-Lys catalytic dyad^12^. Oliveira and Lindblad^17^ reported that LexA in S.6803 exists in at least three forms with different isoelectric points. This may suggest posttranslational control of LexA activity but no information is available regarding the nature of the modification and the conditions under which modification pattern changes.

To clarify the physiological role of cyanobacterial LexA and the regulatory mechanism of its activity, here we focus on salt acclimation of S.6803, since our RNA-seq data revealed that expression levels of a set of genes related to accumulation of the osmoprotective compound glucosylglycerol (GG) were significantly affected in the *lexA*-disrupted mutant^9^. When exposed to salt-stress conditions, S.6803 starts to synthesize GG by a two-step reaction^18^. First GG-phosphate is synthesized from glycerol-3-phosphate and ADP-glucose by GG-phosphate synthase (GgpS) and then GG-phosphate is dephosphorylated by GG-phosphate phosphatase (GgpP) to yield GG. The ABC-type transporter for GG uptake composed of four subunits, GgtA, GgtB, GgtC and GgtD, is also induced upon the salt stress. The main function of this transporter is suggested to be the reuptake of GG leaked into the periplasm to avoid carbon loss^19^. In addition, the GG-degrading enzyme GghA encoded by a salt-inducible gene *slr1670* was supposed to work for GG turnover in fluctuating salinities^20^. In the genome of S.6803, genes encoding these GG-related enzymes and transporters together with genes related to glycerol-3-phosphate metabolism (*glp* genes) are organized into four gene clusters and their expression is highly induced under non-stress conditions by disruption of *lexA*^9^.

In this study, the contribution of LexA to transcriptional regulation of GG-related gene clusters together with other salt stress-inducible genes was evaluated before and after salt stress. Although expression of many salt stress-inducible genes are directly or indirectly repressed by LexA under non-stress conditions, their salt response can be attained without LexA. We suggest that the physiological role of LexA in S.6803 is fine-tuning of gene expression in response to environmental changes. To achieve this, LexA activity is likely to be modulated through posttranslational modification instead of digital switching-off regulation employed by heterotrophic bacteria.

## Results

### Contribution of LexA to transcriptional regulation of four gene clusters involved in accumulation of glucosylglycerol

**Figure 1:**
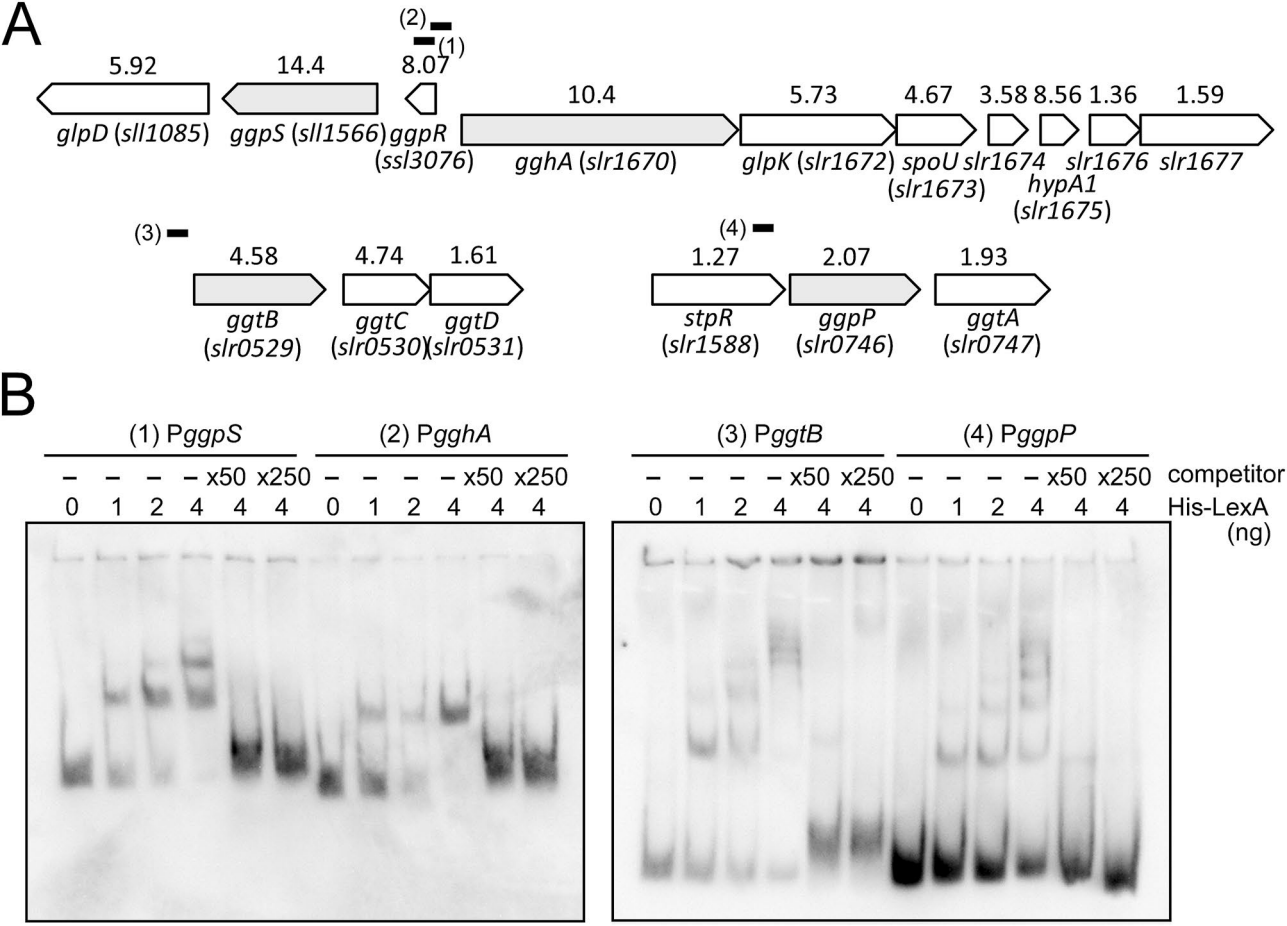
The genomic organization of four gene clusters containing glucosylglycerol biosynthesis- and uptake-related genes in S.6803 and binding of LexA to their upstream region. (**A**) Schematic view of the genomic organization. The first gene of each gene cluster is shown in gray. The numerals above each gene indicate the induction ratio by disruption of *lexA* examined by RNA-seq analysis under non-stress conditions^9^. Short bold bars indicate probes for DNA gel mobility shift assay. (**B**) DIG-labeled promoter segments of four gene clusters, P*ggpS*, P*gghA*, P*ggtB* and P*ggpP*, were incubated with His-LexA added at indicated concentrations. 50-fold and 250-fold excess amounts of the non-labeled promoter segments were added as a competitor. Samples were separated on a 6% polyacrylamide gel.

Figure 1A shows the genomic organization of four gene clusters containing GG biosynthesis- (*ggp*, *glp*) and uptake- (*ggt*) related genes in S.6803. The numerals above each gene indicate the induction ratio by disruption of *lexA* examined by RNA-seq analysis under non-stress conditions^9^. We performed DNA gel mobility shift assays to examine whether all of these four gene clusters were under the direct control of LexA. Approximately 200 bp of DNA fragments containing the upstream promoter region and the major transcription start site determined by differential RNA-seq (dRNA-seq) analysis^21^ were used as probes for the assay (short bold lines in Fig. 1A). The first gene cluster, *ggpS*–*glpD* (*sll1566*–*sll1085*), was reported to have its transcription start site within the upstream small *ggpR* gene encoding a negative regulator of *ggpS* ^22^. Thus, we employed a part of the *ggpR* coding sequence as the P*ggpS* probe. Although *ggpR* was reported to show salt-independent constitutive expression^22^, high induction of *ggpR* transcripts by disruption of *lexA* (Fig. 1A) may imply that *ggpR* is also under the negative control by LexA independent of *ggpS*–*glpD*. The second gene cluster including *gghA* (*slr1670*) and *glpK* (*slr1672*) shares the promoter region with the divergently transcribed *ggpS*–*glpD*. The co-induction of *gghA*–*glpK*–*spoU*–*slr1674*–*hypA1* by disruption of *lexA* (Fig. 1A) suggests polycistronic expression of these five genes. The third and fourth gene clusters are *ggtBCD* (*slr0529*–*0531*) and *ggpP*–*ggtA* (*slr0746*–*0747*). The upstream situated gene of *ggpP*–*ggtA, stpR* (*slr1588*) encoding a negative regulator of *spsA*^23^, was reported to be monocistronically transcribed based on the location of the transcription start sites^21,23^. Thus we used a part of the *stpR* coding sequence as the P*ggpP* probe for DNA gel mobility shift assay. The results of DNA gel mobility shift assay are shown in Fig. 1B. His-LexA recombinant protein bound to the upstream region of all of these gene clusters. The addition of 50-fold and 250-fold excess amounts of the non-labeled specific promoter segments as a competitor abolished the formation of the shifted complex for each probe. These results suggest a mechanism for coordinated transcriptional regulation of GG-related genes by the specific binding of LexA.

In order to examine the role of LexA on salt-induced expression of GG-related genes, the wild-type (WT) strain and the *lexA*-disrupted (Δ*lexA*) mutant were transferred from non-stress to salt-stress conditions by the addition of NaCl. The growth of WT was not delayed even in the presence of 500 mM NaCl which has been generally used as salt stress conditions in S.6803 (Supplementary Fig. S1). The growth of Δ*lexA* was also not affected by NaCl but the mutant grew slower than WT as reported previously^9^.

**Figure 2:**
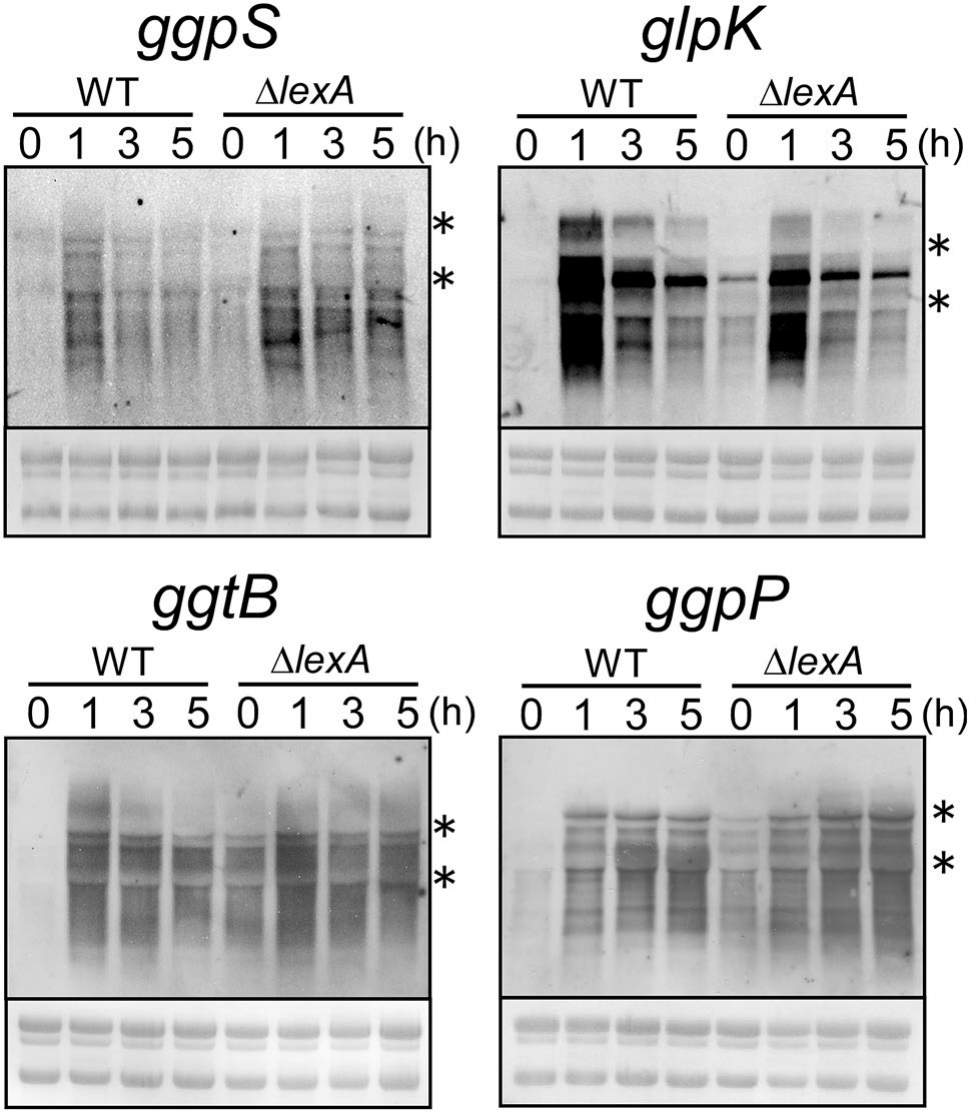
Changes in transcript levels of four GG-related gene clusters upon the addition of 500 mM NaCl in WT and Δ*lexA* strains. Total RNA was isolated from cells before and after the addition of NaCl at time points indicated. 5 μg of total RNA was applied to each lane for RNA gel blot analysis and transcripts were detected by using single-stranded riboprobes of *ggpS*, *glpK*, *ggtB* and *ggpP*. rRNA was visualized with methylene blue staining to show the equal RNA loading. The location of 23S and 16S rRNA was shown in each panel by asterisks.

Changes in transcript levels of GG-related gene clusters before and after the addition of 500 mM NaCl were detected by RNA gel blot analysis using *ggpS*, *glpK*, *ggtB* and *ggpP* probes, respectively (Fig. 2). As reported in the previous studies^19,24^, smearing of the bands was observed, which may be caused by both low stability of GG-related transcripts and existence of multiple transcription units within a gene cluster. Under non-stress conditions (0 h), expression of GG-related transcripts was tightly repressed in WT, whereas substantial expression was observed in the Δ*lexA* mutant, which is consistent with the previous RNA-seq results (Fig. 1A)^9^. Upon the addition of NaCl, GG-related transcripts were highly accumulated within 1 h. Although GG-related genes were derepressed in the Δ*lexA* mutant under non-stress conditions, further upregulation was clearly observed in response to salt stress. This observation suggests that induction of GG-related genes under salt-stress conditions is mainly governed by alternative, unidentified regulators.

**Figure 3:**
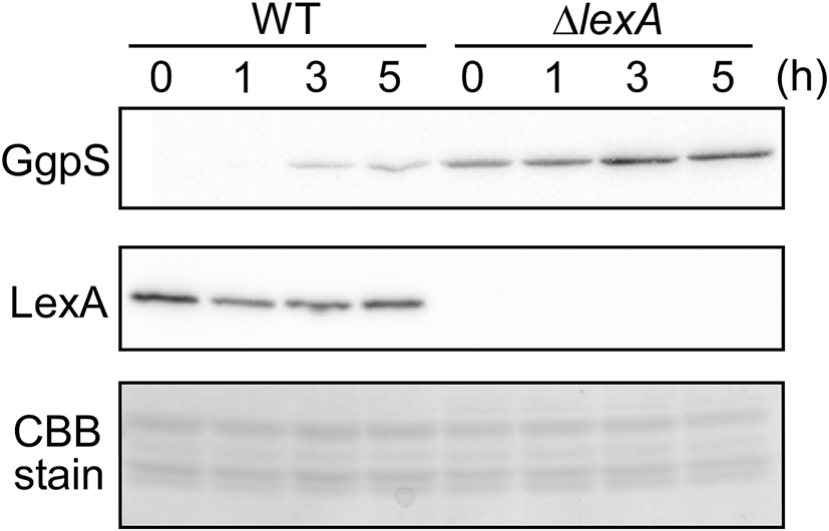
Changes in protein levels of GgpS and LexA upon the addition of 500 mM NaCl in WT and Δ*lexA* strains. Total proteins were isolated from cells before and after the addition of NaCl at time points indicated. 5 μg of total proteins were applied to each lane for immunoblot analysis and detected by specific antibodies. A part of Coomassie Brilliant Blue (CBB) stained SDS-PAGE gel was presented to show the equal protein loading.

The effect of the lack of repression by LexA on GgpS protein level was examined by immunoblot analysis (Fig. 3). The GgpS protein (57 kDa) was detected only after 3 h of the addition of NaCl in WT, whereas it was already accumulated under non-stress conditions and further increased after the addition of NaCl in the Δ*lexA* mutant. The amount of LexA (27 kDa) was almost unchanged before and after the addition of NaCl in WT (Fig. 3). Neither a decrease in the size of LexA nor the appearance of auto-cleavage products were detected.

### Contribution of LexA to transcriptional regulation of salt-stress inducible genes

Previous DNA microarray studies revealed that expression of several hundreds of genes is induced or repressed under salt-stress conditions^25,26^. We wondered whether LexA is involved in regulation of not only GG-related genes but also other salt-stress responsive genes. To evaluate to what extent LexA contributes to salt-stress response, we listed 140 genes reported as NaCl-inducible in at least two of the previous reports^25–28^ in Supplementary Table S1 and added our previously generated RNA-seq data on induction ratio of each gene by disruption of *lexA*^9^. Among 140 NaCl-inducible genes, 63 genes (45%) were induced more than two-fold but no gene was repressed less than half under non-stress conditions by disruption of *lexA*. As for genes whose expression was repressed under salt stress^25^, the effect of disruption of *lexA* on their expression was quite limited (Supplementary Table S2), and the most affected gene among them, *psaD*, has been experimentally shown not to be a direct target of LexA^9^. Taken together, these data suggest the function of LexA as a general repressor of NaCl-inducible genes. Marin et al.^26^ categorized salt-stress inducible genes into four groups according to the time of maximum induction, 15 min, 30 min, 2 h and 6 h. As shown in Supplementary Table S3, highly induced genes by disruption of *lexA* were concentrated into a group with a maximum induction at 2 h, including GG-related genes such as *ggpS*, *gghA* and *glpD*.

**Figure 4:**
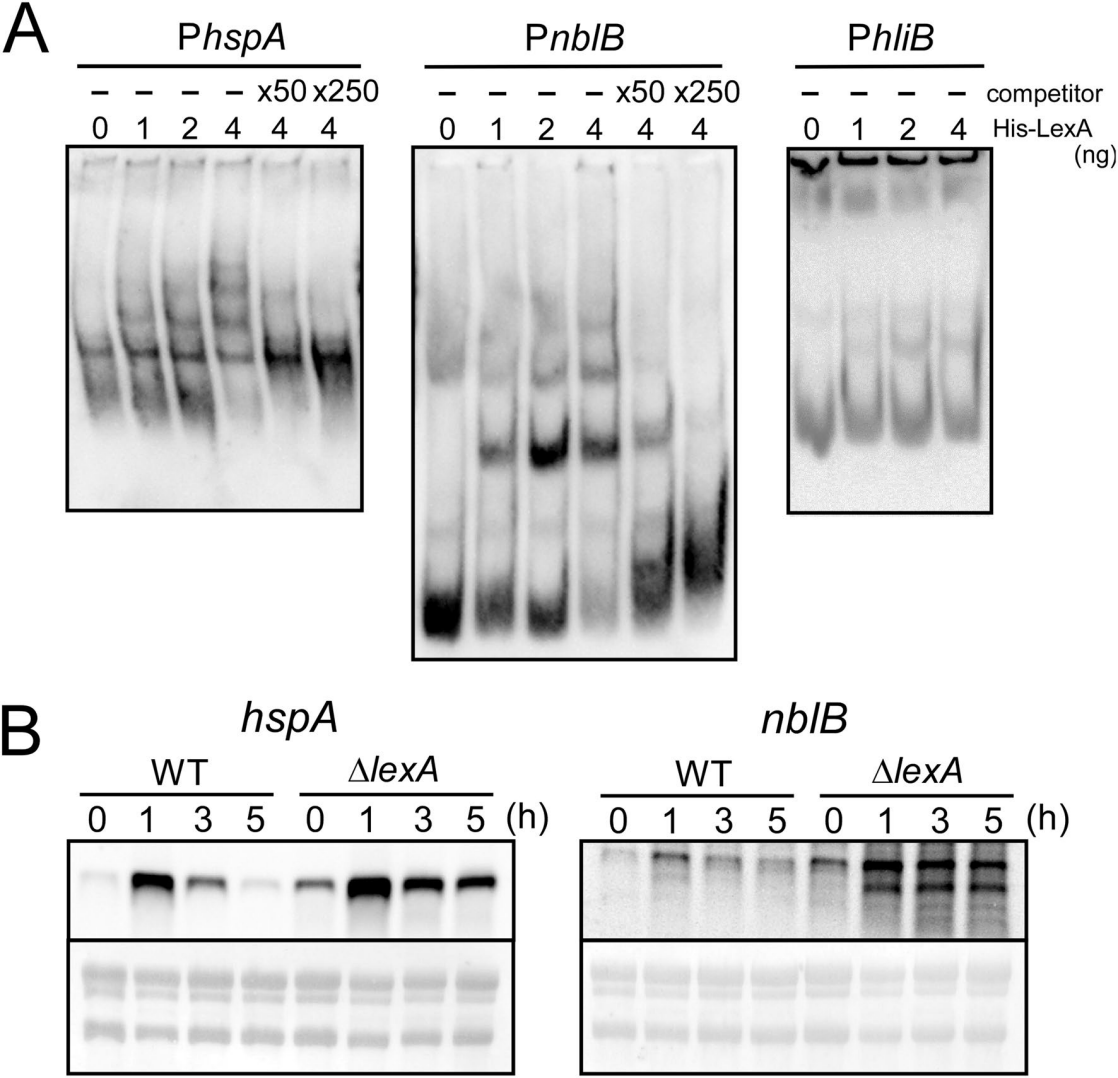
Regulation of salt-stress inducible genes by direct binding of LexA. (**A**) DNA gel mobility shift assay. DIG-labeled promoter segments of *hspA*, *nblB* and *hliB* were incubated with His-LexA added at indicated concentrations. 50-fold and 250-fold excess amounts of the non-labeled promoter segments were added as a competitor. Samples were separated on a 6% polyacrylamide gel. (**B**) RNA gel blot analysis. Total RNA was isolated from WT and Δ*lexA* cells before and after the addition of 500 mM NaCl at time points indicated. 5 μg of total RNA was applied to each lane and transcripts were detected by using single-stranded riboprobes of *hspA* and *nblB.* rRNA was visualized with methylene blue staining to show the equal RNA loading.

To test whether LexA directly regulates expression of other members than GG-related genes in this group, we chose *hspA* (*sll1514*) encoding a small heat-shock protein, *hliB* (*ssr2595*) encoding a high-light inducible protein and *nblB* (*slr1687*) encoding a protein involved in degradation of phycobilisome, and performed DNA gel mobility shift assays using the upstream intergenic region of these genes as DNA probe (Fig. 4A). Specific binding of His-LexA protein to the upstream region of *hspA* and *nblB* was observed, whereas band shift was not detected in the case of *hliB*. When examined by RNA gel blot analysis (Fig. 4B), the expression level of *hspA* and *nblB* under non-stress conditions (0 h) was clearly higher in the Δ*lexA* mutant than in WT. Upon the addition of 500 mM NaCl, the pattern of the salt response was similar between both strains but transcript levels were always higher in the mutant. The presumable degradation product of *nblB* was specifically detected in the mutant under salt stress conditions. These observations suggest that LexA binds to the upstream region of *hspA* and *nblB* and works as repressor irrespective of salt concentration.

**Figure 5:**
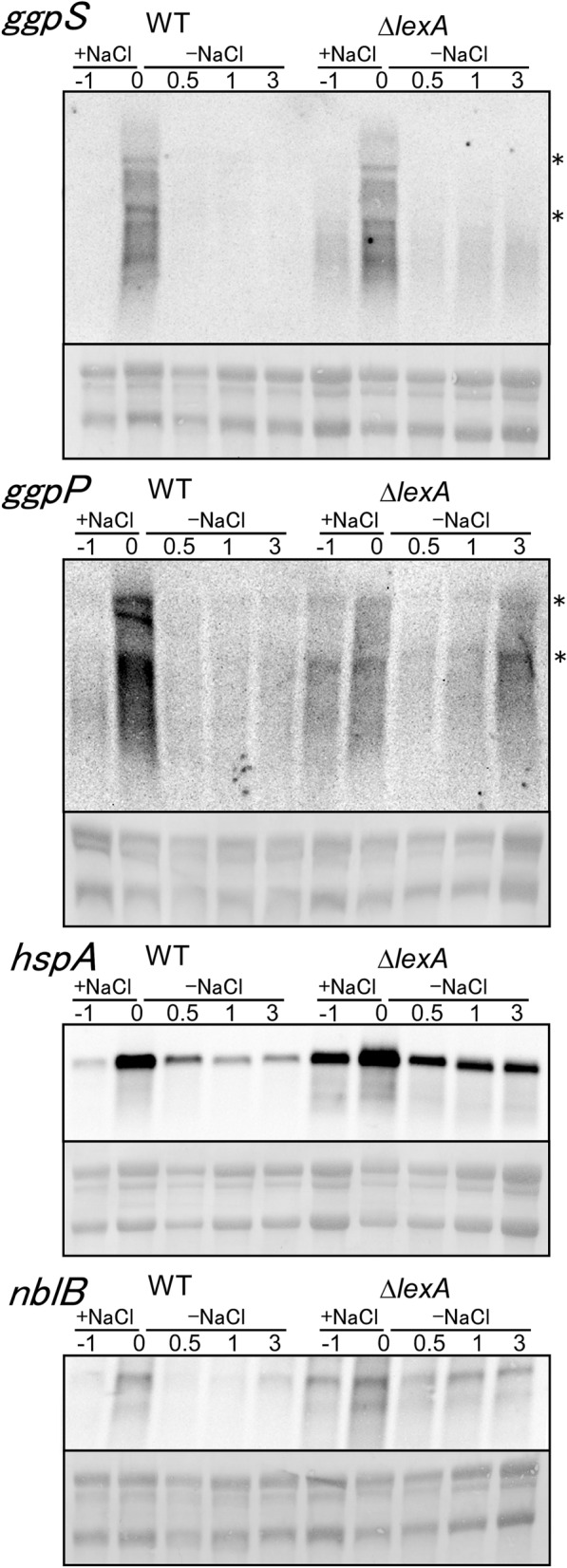
Changes in transcript levels upon the downshift in salt concentration in WT and Δ*lexA* strains. Total RNA was isolated from cells before (− 1 h) and after 1 h of incubation with 500 mM NaCl (0 h) and at time points indicated after transferred to the normal BG-11 medium (0.5, 1 and 3 h). 5 μg of total RNA was applied to each lane for RNA gel blot analysis and transcripts were detected by using single-stranded riboprobes of *ggpS*, *ggpP*, *hspA* and *nblB*. rRNA was visualized with methylene blue staining to show the equal RNA loading. The location of 23S and 16S rRNA was shown by asterisks.

Next, we examined whether LexA is involved in downregulation of salt-stress inducible genes upon transfer from salt stress to non-stress conditions (Fig. 5). Incubation under salt stress conditions for 1 h resulted in accumulation of transcripts of *ggpS*, *ggpP*, *hspA* and *nblB* in both WT and the Δ*lexA* mutant. Upon the down-shift in salt concentration, *ggpS* and *ggpP* transcripts significantly decreased within 30 min in both strains and tended to gradually reaccumulate in the mutant. In the case of *hspA* and *nblB*, transcript levels were always higher in the mutant as observed in the up-shift experiment of salt concentration (Fig. 4B).

### Changes in posttranslational modification of LexA protein during salt stress

**Figure 6:**
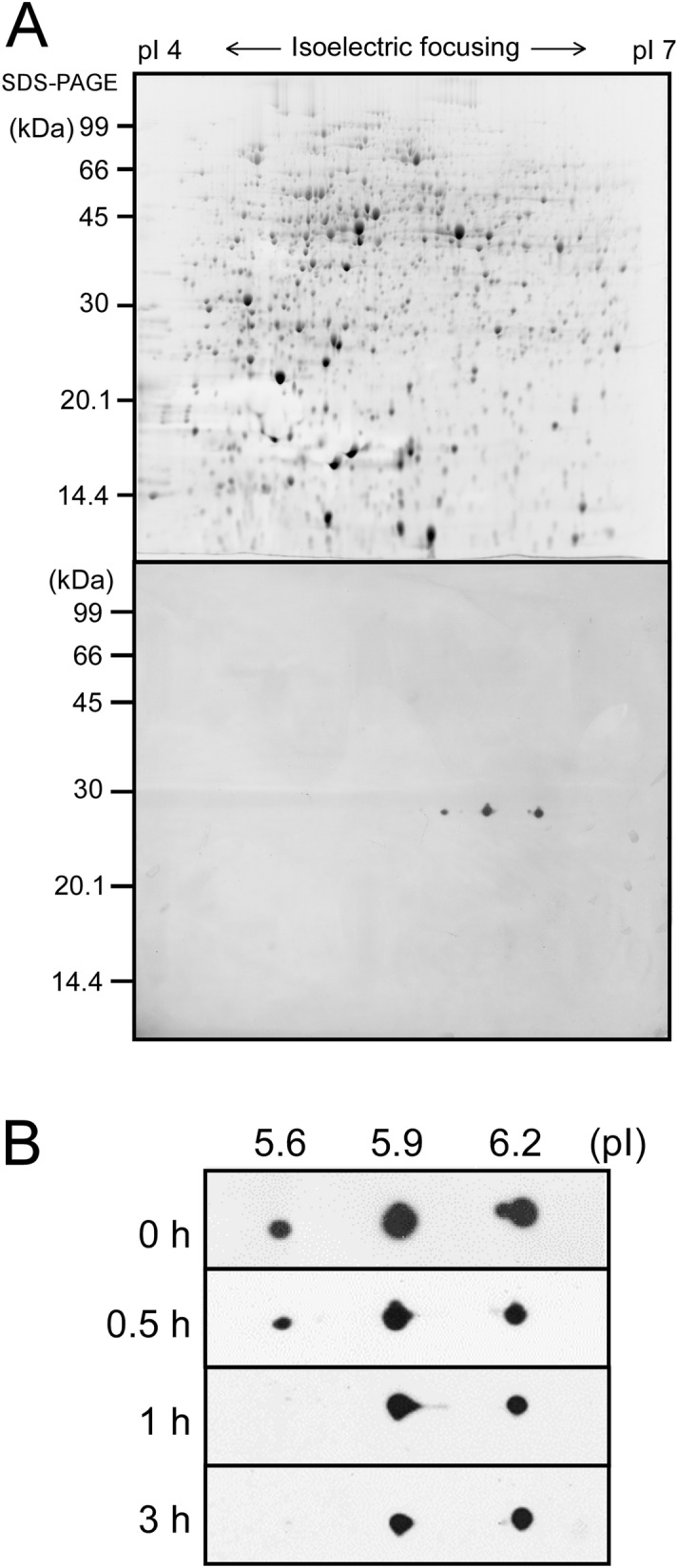
Changes in posttranslational modification of LexA upon the addition of 500 mM NaCl examined by 2D electrophoresis. (**A**) SYPRO Ruby-stained 2D gel with 270 μg of total protein from S.6803 grown under non-stress conditions (upper panel) and immunodetection of LexA using anti-LexA antibody (lower panel). At least three different forms of LexA with pIs of approximately 5.6, 5.9, and 6.2 were detected. (**B**) Changes in the spot pattern of LexA before and after the addition of NaCl at time points indicated.

As shown in Fig. 3, the amount and size of the LexA protein did not change during salt stress. Furthermore, blue native PAGE (BN-PAGE) followed by immunodetection of LexA revealed that the oligomerization state of LexA did not change before and after salt treatment (Supplementary Fig. S2). The detected size was larger than the dimeric form of LexA (54 kDa), which may be due to slower mobility of LexA than the molecular weight marker in BN-PAGE. Since the presence of at least three forms of LexA with different isoelectric points (pIs), 5.6, 5.8 (theoretical pI of LexA) and 6.1 was reported^17^, we performed two-dimensional (2D) electrophoresis to detect changes in posttranslational modification of LexA after 0, 0.5, 1 and 3 h of incubation with NaCl. Immunodetection with anti-LexA antibody revealed three spots with different pIs, 5.6, 5.9 and 6.2 (Fig. 6A), which is consistent with the previous report. The spot with apparent pI of 5.6 disappeared after 30 min, but the modification state of the rest of LexA seemed unchanged during salt treatment (Fig. 6B).

To further test the possibility of regulation of LexA activity by posttranslational modification, we performed immunoprecipitation of LexA from S.6803 cell extracts after 0, 1 and 3 h of incubation with NaCl. After SDS-PAGE of the eluted fraction followed by silver staining (Supplementary Fig. S3), LexA bands were excised from the gel, digested with trypsin, and then analyzed with liquid chromatography-tandem mass spectrometry (LC–MS/MS). Obtained data suggested phosphorylation of several Ser, Thr and Tyr residues in LexA (Supplementary Table S4). In order to evaluate the change in the phosphorylation state of LexA in response to salt stress, the abundance of phosphopeptides was normalized by the abundance of LexA protein recovered from the excised gel and then the abundance ratio of 1 h/0 h and 3 h/0 h was calculated for each phosphopeptide (Supplementary Table S5). The most notable change was a decrease in the abundance ratio of the ASNNKGPGQELKASDVEIQGILMGVWR peptide to 0.26 after 1 h. The phosphorylation site in this peptide was identified as the second Ser (Supplementary Fig. S4), corresponding to Ser^173^ in LexA. However, the decrease in the phosphorylation rate upon salt stress could not be confirmed by the other peptides containing Ser^173^. The ASNNKGPGQELK and ASNNK peptides were not detected as phosphopeptides, probably because of low recovery by the reversed phase chromatography due to their low hydrophobicity. The VVLKASNNKGPGQELK phosphopeptide was detected but a change in the abundance ratio was not clearly observed probably due to its low abundance (Supplementary Table S5). The abundance of the phosphorylated form of the ASNNKGPGQELKASDVEIQGILMGVWR peptide (Supplementary Table S4, lines 50 and 51) was one order lower than that of the non-phosphorylated form (Supplementary Table S4, lines 52 and 54). Taken together, the results of LC–MS/MS analysis suggest that a small fraction of LexA molecules is phosphorylated at Ser^173^ under non-stress conditions and dephosphorylated within 1 h upon the addition of NaCl.

## Discussion

DNA microarray analyses to examine the transcriptional response of S.6803 to salt stress revealed the induction of genes encoding proteins required for high salt acclimation as well as those encoding general stress response proteins^25–28^. By re-analysis of our previously generated RNA-seq data^9^, we found that disruption of *lexA* resulted in induction of about half of these salt-stress inducible genes more than two-fold under non-stress conditions (Supplementary Table S1). DNA gel mobility shift assay revealed that LexA directly regulates all of four gene clusters involved in GG accumulation, as well as general stress responsive genes, *hspA* and *nblB* (Figs. 1B, 4A). These results suggest that LexA works as a general repressor of salt-inducible genes. However, induction of these genes upon salt stress and repression upon transfer to normal growth conditions took place irrespective of the presence or absence of LexA (Figs. 2, 4B, 5). Changes in activity of transcription factors other than LexA and/or stability of transcripts may be mainly responsible for salt stress responses of these genes. Shoumskaya et al.^28^ reported that several two-component systems, namely the pairs of histidine kinase and response regulator, Hik33–Rre31, Hik10–Rre3, Hik16–Hik41–Rre17 and Hik34–Rre1, are involved in salt-induction of certain subsets of genes in response to various stresses indirectly caused by the addition of salt, such as generation of reactive oxygen species, redox imbalance and denatured proteins. In that study, *hspA* and *nblB* were suggested to be under the control of Rre1 and Rre31, respectively. There have been no reports of identification of regulators other than LexA involved in the coordinated regulation of all of the four GG-related gene clusters. As for *ggpS*, several studies suggest the existence of specific regulators. For example, GgpR encoded by *ssl3076*, a small ORF adjacent to *ggpS*, works as a repressor of *ggpS*^22^, the group 2 sigma factor SigB affects the timing of induction of *ggpS* upon salt stress^24^ and small RNA IsaR1 interacts with the 5′-UTR of *ggpS* to negatively affect its stability and translation^29^. Salt response of the other GG-related genes may also be attained by specific regulators not yet identified.

Based on the observation in this study, we propose that the contribution of LexA to salt acclimation is not induction of salt-stress responsive genes but optimization of energy distribution by repressing unnecessary transcription. In the Δ*lexA* mutant, the salt-stress responsive genes were derepressed under non-stress conditions (Figs. 2, 4B, Supplementary Table S1) and their induction levels upon salt stress were sometimes higher than WT (Figs. 2, 4B). Accumulation of GgpS protein under non-stress conditions was also observed in the mutant (Fig. 3). Considering that LexA is a global regulator involved in regulation of various cellular processes^9^, such uncontrolled expression of the LexA regulon must cause energy losses and delayed growth of the mutant both under normal and salt-stress conditions (Supplementary Fig. S1).

In heterotrophic bacteria such as *E. coli*, activity of LexA is controlled by digital switching-off mechanism through auto-cleavage in response to DNA damage. On the other hand, there have been no reports on physiological changes in the amount and oligomerization state as well as auto-cleavage in the case of cyanobacterial LexAs. In this study, we first expected switching-off of repressing activity of LexA upon salt stress. However, induction levels of some salt-responsive genes such as *ggpS*, *hspA* and *nblB* were always higher in the Δ*lexA* mutant than in WT (Figs. 2, 4B, 5), suggesting that repressing activity of LexA can persist during salt stress. We propose here that activity of LexA in S.6803 is not controlled in a digital ON–OFF manner in response to environmental changes, but there may exist gradual control through posttranslational modification.

We observed disappearance of the LexA spot with an apparent pI of 5.6 after 30 min of salt-treatment by 2D electrophoresis (Fig. 6B) and a decrease in the phosphorylation level of Ser^173^ within 1 h by immunoprecipitation followed by LC–MS/MS analysis (Supplementary Table S5). It is worth noting that both the pI 5.6 spot and phosphorylated Ser^173^ represent a minor fraction of the total LexA population. Upon salt acclimation, a large fraction of LexA may maintain the activity as a repressor but dephosphorylation of Ser^173^ after 30 min may cause decrease in the activity of some LexA molecules. This heterogeneity in LexA activity could explain the difference in the repressing effect of LexA among individual target genes (Figs. 2, 4B). Previously, we reported that the repressor activity of LexA on the fatty-acid biosynthetic genes, *fabD*, *fabH* and *fabF*, is enhanced under nitrogen-depleted conditions, and weakened under phosphate-depleted conditions^14^. Examination of changes in 2D spot pattern and phosphorylation level of Ser^173^ under different nutrient conditions will strengthen the view that LexA activity is modulated by posttranslational modification in S.6803.

## Conclusions

We have revealed that LexA in S*.*6803 works as a general repressor of salt-inducible genes but is not the primary regulator of their salt response. Based on the observation, we hypothesize that the contribution of LexA to salt acclimation is optimization of energy distribution by repressing unnecessary transcription. In contrast to the digital switching-off regulation of LexA activity by auto-cleavage in heterotrophic bacteria, activity of LexA in S.6803 may be under more gradual control through posttranslational modification. It seems reasonable to employ such a regulatory mechanism assuming that physiological roles of LexA in S.6803 is fine-tuning of expression levels of genes related to various cellular processes in response to environmental changes. It is worth noting that Ser^173^ is not so much conserved, 26 out of 152 amino acid sequences of cyanobacterial LexAs according to our preliminary search (not shown). This may indicate the diversity in the function and regulatory mechanism of cyanobacterial LexAs. Based on the computational analysis of LexA binding sites across a large number of cyanobacterial genomes, Li et al.^30^ proposed the possibility that LexAs in most cyanobacteria can work as a regulator of SOS response. To obtain the comprehensive picture of the physiological role of cyanobacterial LexAs, we are now examining the distribution of the Ala-Gly auto-cleavage site, the Ser-Lys catalytic dyad, the putative phosphorylation site Ser^173^ and the *recA* gene with or without LexA binding sites throughout the phylogenetic tree of cyanobacteria.

## Materials and methods

### Strains and culture conditions

A glucose-tolerant non-motile strain (GT strain) of *Synechocystis* sp. PCC 6803 was grown at 32 °C in BG-11 medium containing 20 mM HEPES–NaOH, pH7.0, under continuous illumination at 20 μmol photons m^−2^ s^–1^ with bubbling of air. The *lexA* (*sll1626*)-disrupted mutant (Δ*lexA*) was generated by insertion of a kanamycin resistance cassette at nucleotide 1,319,123 of the coding region of *lexA*^9^ and grown under the same conditions as the wild-type cells, except that 20 μg mL^–1^ kanamycin was added to the medium. Cell density was estimated by measuring OD_730_ using a spectrophotometer (model UV-1800, Shimadzu). To perform salt stress experiments, WT and the Δ*lexA* cells grown in BG-11 medium to OD_730_ = 0.8 under non-stress conditions were diluted to OD_730_ = 0.3 and a solution of 3 M NaCl dissolved in BG-11 medium was added to 50 mL culture to give a final concentration of 500 mM. To perform downshift experiments of salt concentration, WT and the Δ*lexA* cells at OD_730_ = 0.3 incubated in BG-11 medium containing 500 mM NaCl for 1 h were harvested by centrifugation, washed with the normal BG-11, and resuspended with 50 mL of the same medium.

### DNA gel mobility shift assay

Probes for DNA gel mobility shift assays were obtained by PCR amplification with primers shown in Supplementary Table S6 using genomic DNA as a template. The 3′ end of the DNA fragment for each probe was labeled with DIG-ddUTP using the terminal transferase method according to the manufacturer's instructions (DIG Gel Shift Kit, second generation; Roche). Overexpression and purification of the recombinant LexA protein with an N-terminal 6 × His-tag and DNA gel mobility shift assays by using a DIG Gel Shift Kit were performed as described in Kizawa et al.^9^.

### RNA gel blot analysis

Isolation of total RNA by the hot phenol method and RNA gel blot analyses, using DIG RNA Labeling and Detection Kit (Roche), were performed as described previously^31^. Template DNA fragments for in vitro transcription to generate RNA probes were prepared by PCR using the primers shown in Supplementary Table S6. Original uncropped images of RNA Gel Blot Analysis were shown in Supplementary Fig. S5.

### Immunoblot analysis

Total proteins were extracted from *Synechocystis* cells as described previously^32^ and separated by 15% (w/v) SDS-PAGE, followed by electroblotting onto PVDF membranes (Immobilon-P; Millipore). Immunodetection was done using a rabbit polyclonal antibody raised against the recombinant proteins in combination with a horseradish peroxidase-conjugated secondary antibody. Antibodies raised against GgpS were kindly provided by Prof. M. Hagemann (Universität Rostock). Original uncropped images of Immunoblot Analysis were shown in Supplementary Fig. S5.

### BN-PAGE

Total proteins were extracted from *Synechocystis* cells as described previously^32^. Samples were added with 5 × BN-PAGE sample buffer, containing 240 mM Tris–HCl, pH8.0, 250 mM 6-amino caproic acid, 30% (v/v) glycerol, 0.57 M sucrose and 50 mg mL^−1^ Coomassie G-250. 10 μg protein per lane was applied to 5–20% native-PAGE gel (Kitasato Medical Service) and run according to the manufacturer’s instructions. After incubating the gels in resolubilization buffer containing 0.3% (w/v) Tris, 0.7% (w/v) Glycine and 0.2% (w/v) SDS, electroblotting and immunodetection of LexA were performed as described above.

### 2D gel electrophoresis

For 2D analysis, 350 mL of WT culture at OD_730_ = 0.5 was exposed to salt stress and harvested by centrifugation. The cell pellet was resuspended in 400 μL of 10 mM HEPES–NaOH buffer, pH7.0, containing 10 mM PMSF, supplemented with 10 mM NaF and 1 mM Na_3_VO_4_^33^ and mixed with 3.6 g of zircon beads (diameter 0.1 mm, BioSpec Products) in a 2 mL tube. The cells were then disrupted with a Mini-Bead Beater (BioSpec Products) for three pulses of 50 s at 4 °C. After the cell debris was pelleted by centrifugation, the soluble protein fraction was obtained by ultracentrifugation at 100,000 g for 1 h at 4 °C, quantified by the Bio-Rad protein assay (Bio-Rad), and then concentrated by acetone precipitation. The dried pellet was resuspended with extraction buffer containing 8 M urea, 2 M thiourea, 1% (w/v) CHAPS, 19.4 mM dithiothreitol, and 0.5% Ampholytes (GE Healthcare) and protein concentration was adjusted to 15 mg mL^−1^. For two-dimensional analyses, 270 μg of total protein resuspended with extraction buffer was absorbed by 18-cm-long Immobiline DryStrip gels (GE Healthcare) with a pH range of 4 to 7 (linear), according to the manufacturer's instructions. The isoelectric-focusing step was carried out on CoolPhorestar IPG-IEF Type-PX system (Anatech), followed by 15% (w/v) SDS-PAGE in the second dimension. After completion of SDS-PAGE, total proteins on the gels were stained with SYPRO Ruby (Thermo Fisher Scientific) and images of the SYPRO Ruby-stained gels were acquired using FluoroPhorester 3000 (Anatech). After washing of the gels for 30 min twice with water and then incubating for 30 min in resolubilization buffer containing 0.3% (w/v) Tris, 0.7% (w/v) glycine and 0.2% (w/v) SDS, electroblotting and immunodetection of LexA were performed as described above.

### Immunoprecipitation of LexA protein

For immunoprecipitation, 50 mL of WT culture at OD_730_ = 0.5 was exposed to salt stress and harvested by centrifugation. The cell pellet was resuspended in 500 μL of lysis buffer containing 50 mM HEPES–KOH, pH7.5, 140 mM NaCl, 1 mM EDTA, 1% Triton X-100 and 0.1% sodium deoxycholate, supplemented with Complete Mini EDTA-free protease inhibitor cocktail (Roche). Cells were added with 0.5 g of zircon beads (BioSpec Products), broken by vigorous vortexing, centrifuged at 14,000 g at 4 °C for 15 min and the supernatant was collected as the cell lysate for immunoprecipitation. After determination of the protein concentration using the Bio-Rad protein assay, the cell lysate containing 500 μg of protein was brought to a volume of 500 μL with cold lysis buffer, added with 20 μL of the pre-equilibrated Dynabeads Protein G (Thermo Fisher Scientific) and rotated for 1 h at 4 °C to avoid non-specific binding during the immunoprecipitation step. After removal of Dynabeads Protein G by magnetic separation, the cell lysate was added with 80 μg of anti-LexA antibody and rotated at 4 °C for overnight. Then 20 μL of the equilibrated Dynabeads Protein G were added and rotated at 4 °C for 1 h. After the binding reaction, Dynabeads Protein G was collected by magnetic separation, washed with 1.5 mL of lysis buffer by rotating at 4 °C for 5 min and again collected by magnetic separation. The washing steps were repeated sequentially using 1 mL of the following buffers: wash buffer1 (lysis buffer containing 500 mM NaCl), wash buffer 2 (10 mM Tris–HCl, pH8.0, 250 mM LiCl, 0.5% NP-40, 0.5% sodium deoxycholate, 1 mM EDTA) and TE (10 mM Tris–HCl, pH8.0, 1 mM EDTA). Elution was performed by incubation with 50 μL of elution buffer (1% SDS, 0.1 mM NaHCO_3_) at room temperature for 15 min with occasional vortexing. The supernatant was collected and the elution step was repeated once. Total 100 μL of eluates were subjected to SDS-PAGE and 27 kDa bands of LexA protein were excised after silver staining.

### LC–MS/MS analysis

The silver-stained bands of LexA were de-stained and digested with a trypsin (TPCK-treated; Worthington Biochemical). The digestion mixture was separated on a nanoflow LC (Easy nLC; Thermo Fisher Scientific) using a nano-electrospray ionization spray column (NTCC analytical column; C18, φ75 μm × 100 mm, 3 μm; Nikkyo Technology) with a linear gradient of 0–80% buffer B (100% acetonitrile and 0.1% formic acid) in buffer A (0.1% formic acid) and a flow rate of 300 nL min^−1^ over 30 min, coupled on-line to a Q-Exactive mass spectrometer (Thermo Fisher Scientific) that was equipped with a nanospray ion source, based on the method described in Nagao et al.^34^. The mass spectrometer was operated in positive-ion mode, and MS and MS/MS spectra were acquired in a data dependent TOP 10 method. Protein were identified and quantified using Proteome Discoverer 2.2 (Thermo Fisher Scientific) with MASCOT program ver 2.6 (Matrix Science).

## Supplementary information

Supplementary Tables.

Supplementary Figures.
