# Supplementary material for: The role of transcriptional repressor activity of LexA in salt-stress responses of the cyanobacterium Synechocystis sp. PCC 6803
Source: Sci Rep. 2020 Oct 15;10:17393. doi: 10.1038/s41598-020-74534-7 (PMC7567884; doi:10.1038/s41598-020-74534-7)
Supplement: Supplementary file 2 — Supplementary Figures. [file 41598_2020_74534_MOESM2_ESM.pdf]

The role of transcriptional repressor activity of LexA in salt-stress responses of the cyanobacterium *Synechocystis* sp. PCC 6803

Kosuke Takashima[a], Syota Nagao[a], Ayumi Kizawa[a], Takehiro Suzuki[b], Naoshi Dohmae[b], Yukako Hihara[a]*

[a]Graduate School of Science and Engineering, Saitama University, 255 Shimo-Okubo, Saitama 338-8570, Japan
[b]Biomolecular Characterization Unit, RIKEN Center for Sustainable Resource Science, 2-1 Hirosawa, Wako, Saitama 351-0198, Japan

*Corresponding author

Supplementary information

Supplementary Figures: this file
Supplementary Tables: provided separately as spreadsheets (.xlsx)

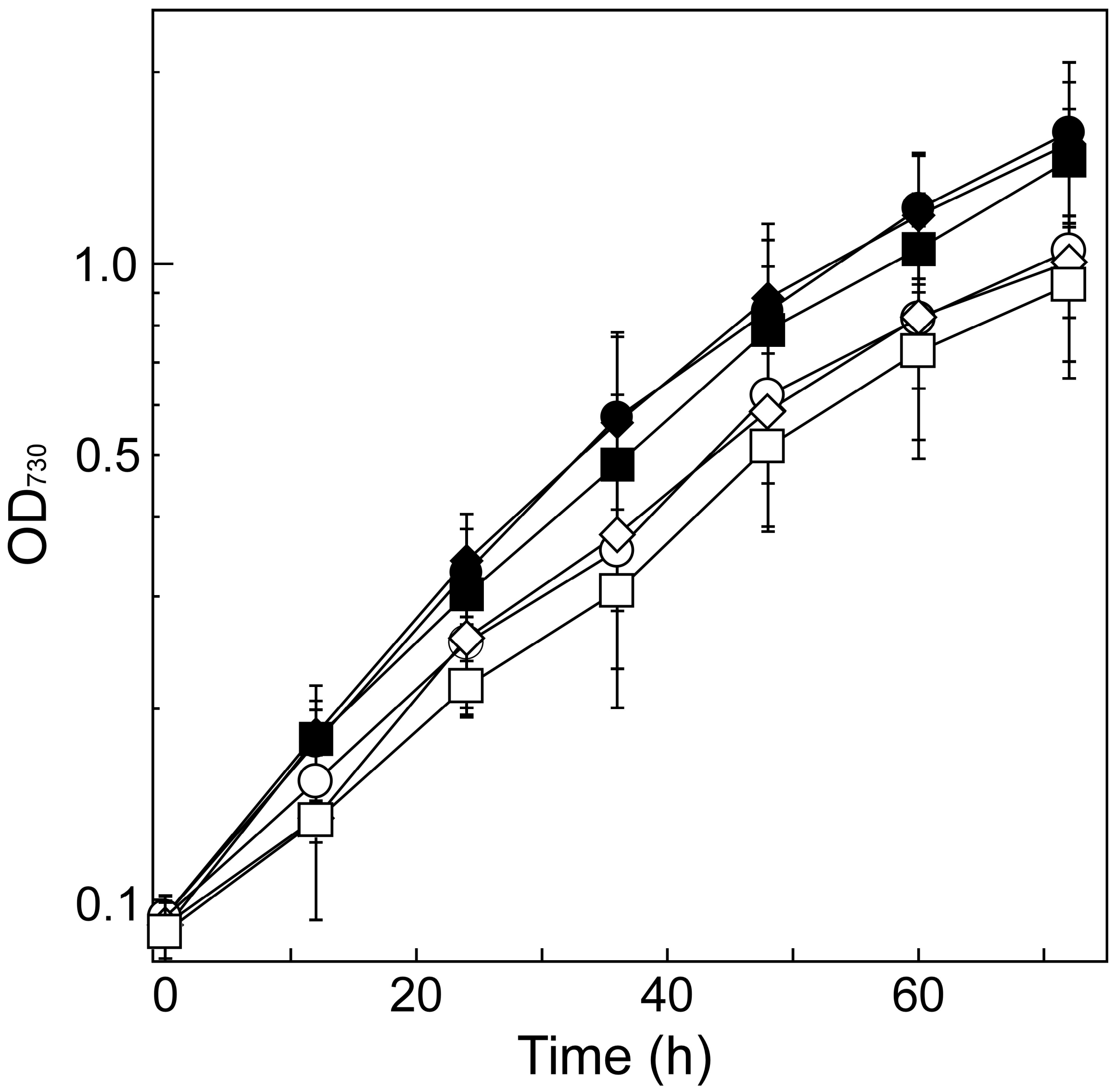

Supplementary Figure S1. Effect of the addition of NaCl on the growth of WT and the Δ*lexA* cells. Cells that had been grown to OD_{730} = 0.5 in normal BG-11 medium were seeded at OD_{730} = 0.1 in normal BG-11 medium (WT:●, Δ*lexA*:○) or in BG-11 medium supplemented with NaCl to the final concentration of 250 mM (WT:◆, Δ*lexA*:◇) or 500 mM (WT:■, Δ*lexA*:□) and grown for 72 h. The data are the mean values ±SD of three biological replicates.

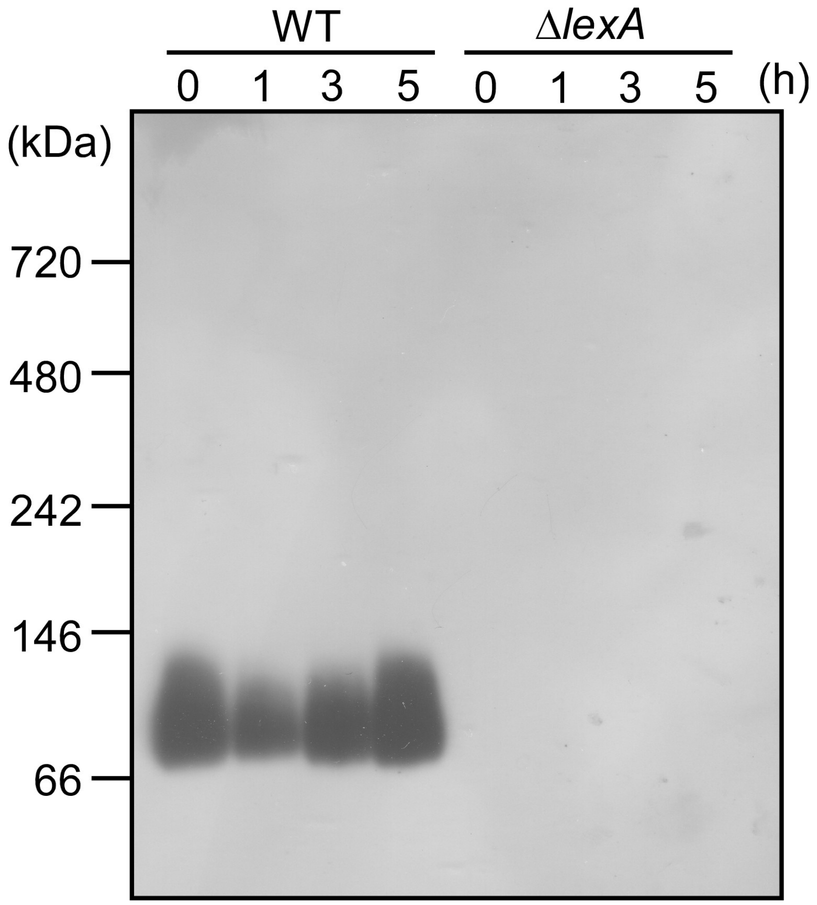

Supplementary Figure S2. Oligomerization state of LexA examined by BN-PAGE of the cell lysate followed by immunodetection of LexA. The cell lysates of WT and the Δ*lexA* cells were subjected to BN-PAGE, followed by electroblotting onto PVDF membrane and immunodetection using the specific antibody against LexA. Numbers indicate the migration pattern of NativeMark Unstained Protein Standard (Thermo Fisher).

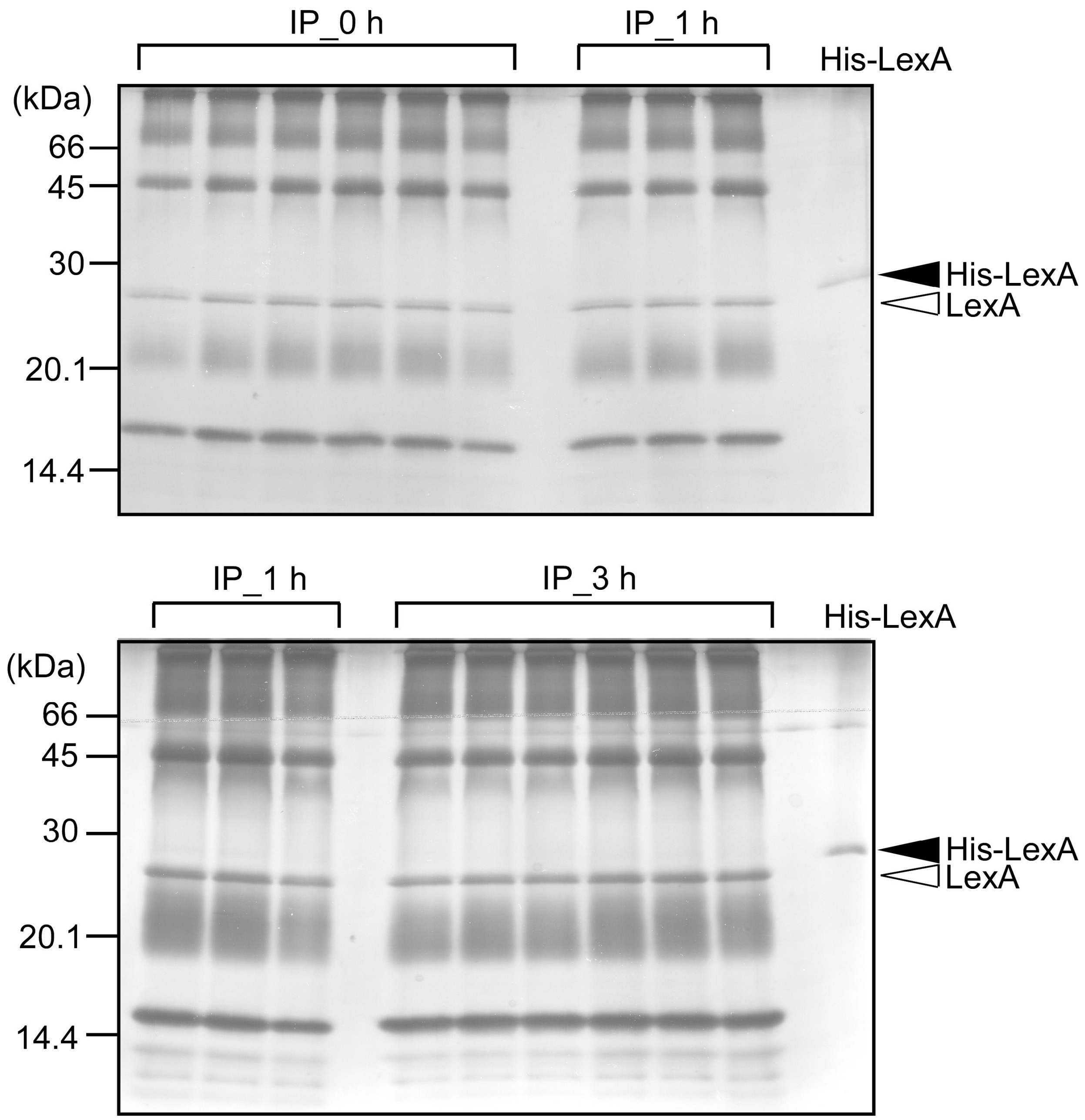

Supplementary Figure S3. Preparative electrophoresis of immunoprecipitated LexA for LC-MS/MS analysis. LexA was immunoprecipitated from S.6803 cell extracts after 0, 1 and 3 h of incubation with 500 mM NaCl. 100 μL of eluate from each time point was divided into six aliquots and subjected to SDS-PAGE. 27 kDa bands of LexA protein (white arrowhead) were excised after silver staining to be analyzed by LC-MS/MS.

MS/MS Fragmentation of ASNNKGPGQELKASDVEIQGILMGVWR
Found in BAA17770.1 in S_PCC6803, SOS function regulatory protein [Synechocystis sp. PCC 6803]

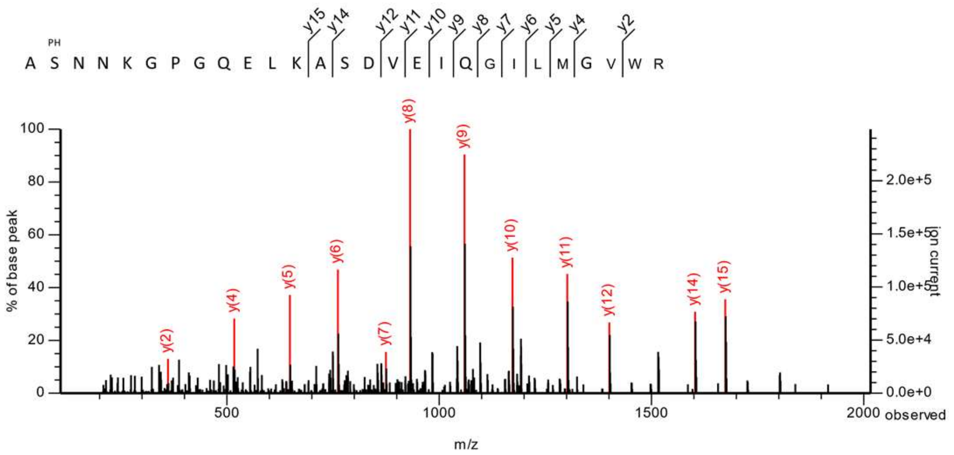

Monoisotopic mass of neutral peptide Mr(calc): 2976.4528

Variable modifications:

S2 : Phospho (ST), with neutral losses 0.0000(shown in table), 97.9769

Ions Score: 86 **Expect:** 2.9e-008

Matches : 12/440 fragment ions using 18 most intense peaks (help)

#	b	b^{++}	b*	b^{*++}	b^0	b^{0++}	Seq.	y	y^{++}	y*	y^{*++}	y^0	y^{0++}	#
1	72.0444	36.5258					A							27
2	239.0427	120.025			221.0322	111.0197	S	2906.423	1453.715	2889.397	1445.202	2888.413	1444.71	26
3	353.0857	177.0465	336.0591	168.5332	335.0751	168.0412	N	2739.425	1370.216	2722.398	1361.703	2721.414	1361.211	25
4	467.1286	234.0679	450.1021	225.5547	449.118	225.0627	N	2625.382	1313.195	2608.355	1304.681	2607.371	1304.189	24
5	595.2236	298.1154	578.197	289.6021	577.213	289.1101	K	2511.339	1256.173	2494.312	1247.66	2493.328	1247.168	23
6	652.245	326.6262	635.2185	318.1129	634.2345	317.6209	G	2383.244	1192.126	2366.217	1183.612	2365.233	1183.12	22
7	749.2978	375.1525	732.2712	366.6393	731.2872	366.1473	P	2326.222	1163.615	2309.196	1155.102	2308.212	1154.61	21
8	806.3193	403.6633	789.2927	395.15	788.3087	394.658	G	2229.17	1115.088	2212.143	1106.575	2211.159	1106.083	20
9	934.3778	467.6926	917.3513	459.1793	916.3673	458.6873	Q	2172.148	1086.578	2155.122	1078.064	2154.138	1077.572	19
10	1063.42	532.2139	1046.394	523.7006	1045.41	523.2086	E	2044.09	1022.548	2027.063	1014.035	2026.079	1013.543	18
11	1176.505	588.7559	1159.478	580.2426	1158.494	579.7506	L	1915.047	958.0271	1898.02	949.5139	1897.036	949.0218	17
12	1304.6	652.8034	1287.573	644.2901	1286.589	643.7981	K	1801.963	901.4851	1784.936	892.9718	1783.952	892.4798	16
13	1375.637	688.3219	1358.61	679.8086	1357.626	679.3166	A	***1673.9***	837.4376	1656.841	828.9243	1655.857	828.4323	15
14	1462.669	731.8379	1445.642	723.3247	1444.658	722.8327	S	***1602.8***	801.9191	1585.804	793.4058	1584.82	792.9138	14
15	1577.696	789.3514	1560.669	780.8381	1559.685	780.3461	D	1515.799	758.403	1498.772	749.8898	1497.788	749.3978	13
16	1676.764	838.8856	1659.737	830.3723	1658.753	829.8803	V	***1400.8***	700.8896	1383.745	692.3763	1382.761	691.8843	12
17	1805.807	903.4069	1788.78	894.8936	1787.796	894.4016	E	***1301.7***	651.3554	1284.677	642.8421	1283.693	642.3501	11
18	1918.891	959.9489	1901.864	951.4357	1900.88	950.9437	I	***1172.7***	586.8341	1155.634	578.3208			10
19	2046.949	1023.978	2029.923	1015.465	2028.939	1014.973	Q	***1059.6***	530.292	1042.55	521.7788			9
20	2103.971	1052.489	2086.944	1043.976	2085.96	1043.484	G	***931.52***	466.2627	914.4917	457.7495			8
21	2217.055	1109.031	2200.028	1100.518	2199.044	1100.026	I	***874.5***	437.752	857.4702	429.2387			7
22	2330.139	1165.573	2313.112	1157.06	2312.128	1156.568	L	***761.41***	381.21	744.3861	372.6967			6
23	2461.179	1231.093	2444.153	1222.58	2443.169	1222.088	M	***648.33***	324.668	631.3021	316.1547			5
24	2518.201	1259.604	2501.174	1251.091	2500.19	1250.599	G	***517.29***	259.1477	500.2616	250.6344			4
25	2617.269	1309.138	2600.243	1300.625	2599.259	1300.133	V	460.2667	230.637	443.2401	222.1237			3
26	2803.349	1402.178	2786.322	1393.665	2785.338	1393.173	W	***361.2***	181.1028	344.1717	172.5895			2
27							R	175.119	88.0631	158.0924	79.5498			1

All matches to this query

Score	Mr(calc)	Delta	Sequence	Site Analysis
85.8	2976.453	-0.0208	ASNNKGPGQELKASDVEIQGILMGVWR	Phospho S2 99.84%
57.9	2976.453	-0.0208	ASNNKGPGQELKASDVEIQGILMGVWR	Phospho S14 0.16%

Supplementary Figure S4. Identification of the phosphorylation site in the phosphopeptide ASNNKGPGQELKASDVEIQGILMGVWR detected by LC-MS/MS analysis.

The phosphorylation analysis was performed by MASCOT program ver 2.6 (Matrix Science) according to the method described in Savitski et al. (2011) Confident phosphorylation site localization using the Mascot delta score. Mol Cell Proteomics 10: M110.003830.

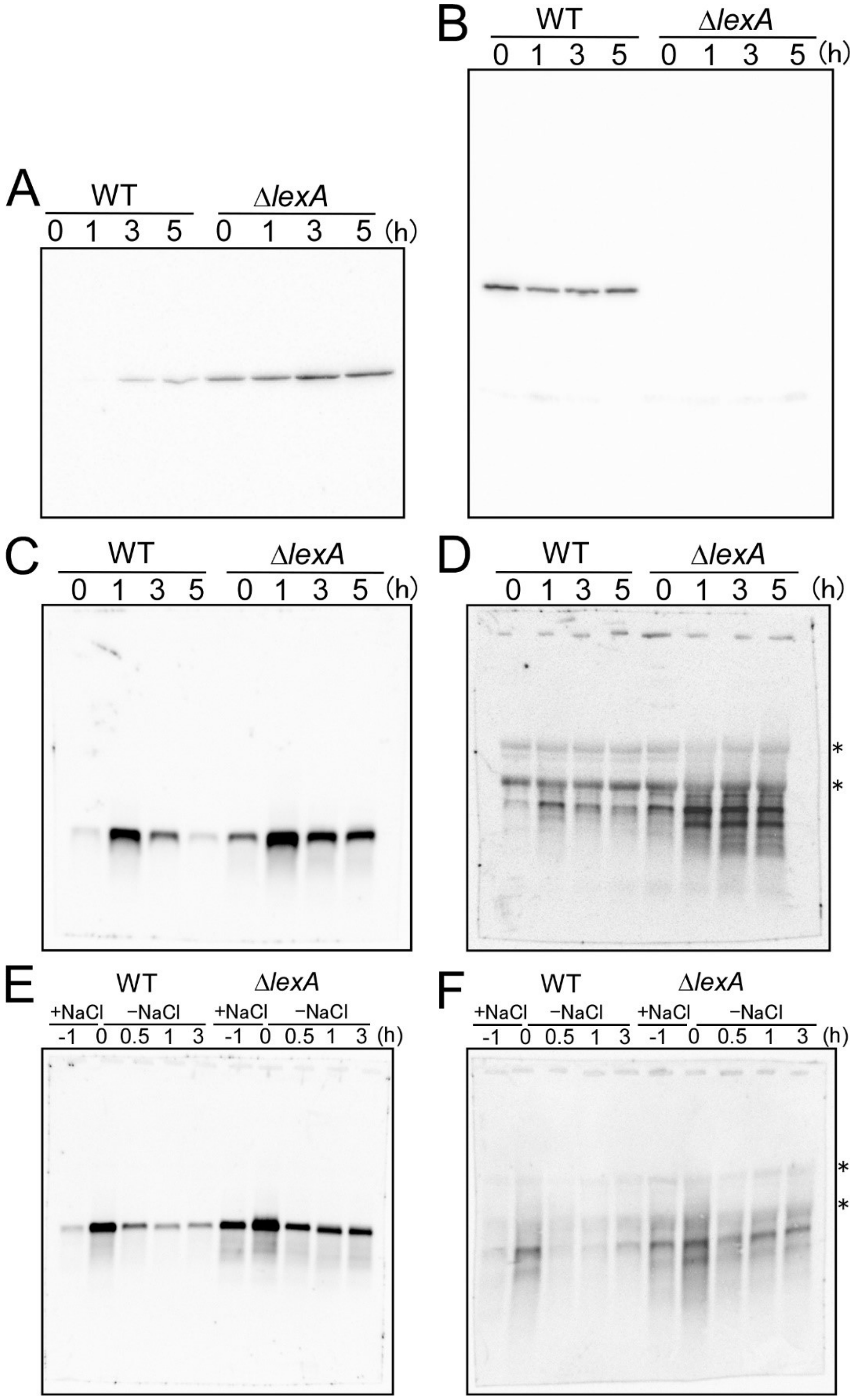

Supplementary Figure S5. Uncropped images of Figures 3, 4B and 5.
A, Figure 3: GgpS. B, Figure 3: LexA. C, Figure 4B: *hspA*. D, Figure 4B: *nblB*, E, Figure 5: *hspA*. F, Figure 5: *nblB*. The asterisks indicate nonspecific hybridization to 23S and 16S rRNAs.
